# Doxycycline-Regulated 3T3-L1 Preadipocyte Cell Line with Inducible, Stable Expression of Adenoviral E4orf1 Gene: A Cell Model to Study Insulin-Independent Glucose Disposal

**DOI:** 10.1371/journal.pone.0060651

**Published:** 2013-03-27

**Authors:** Rashmi Krishnapuram, Emily J. Dhurandhar, Olga Dubuisson, Vijay Hegde, Nikhil V. Dhurandhar

**Affiliations:** Infections and Obesity Laboratory, Pennington Biomedical Research Center, Baton Rouge, Louisiana, United States of America; Tohoku University, Japan

## Abstract

Impaired glycemic control and excessive adiposity are major risk factors for Type 2 Diabetes mellitus. In rodent models, Ad36, a human adenovirus, improves glycemic control, independent of dietary fat intake or adiposity. It is impractical to use Ad36 for therapeutic action. Instead, we identified that *E4orf1* protein of Ad36, mediates its anti-hyperglycemic action independent of insulin signaling. To further evaluate the therapeutic potential of *E4orf1* to improve glycemic control, we established a stable 3T3-L1 cell system in which *E4orf1* expression can be regulated. The development and characterization of this cell line is described here. Full-length adenoviral-36 *E4orf1* cDNA obtained by PCR was cloned into a tetracycline responsive element containing vector (pTRE-Tight-*E4orf1*). Upon screening dozens of pTRE-Tight-*E4orf1* clones, we identified the one with the highest expression of *E4orf1* in response to doxycycline treatment. Furthermore, using this inducible system we characterized the ability of *E4orf1* to improve glucose disposal in a time dependent manner. This stable cell line offers a valuable resource to carefully study the novel signaling pathways *E4orf1* uses to enhance cellular glucose disposal independent of insulin.

## Introduction

Insulin resistance or Type 2 Diabetes mellitus (T2DM) are often associated with impaired insulin signaling [1], [2], [3]. However, most of the currently available anti-diabetic drugs depend on insulin signaling, which may be impaired. Hence, anti-diabetic drugs that act partially or completely independent of insulin signaling may be more effective and desirable. Ad36, a human adenovirus increases cellular glucose uptake and improves hyperglycemia in mice fed a high fat (HF) diet[4] and this action of Ad36 appears to be independent of proximal insulin signaling[5]. Our recent data indicate *E4orf1* transcribed from the first open reading frame of Ad36 early gene 4 is necessary and sufficient for Ad36-induced effect on glucose disposal. Ad36*E4orf1* increases cellular glucose uptake in pre-adipocytes, adipocytes, and myoblasts, and suppression of glucose output by hepatocytes [6], [7]. Therefore *E4orf1* may offer a novel template to develop better anti-diabetic drugs and is currently under investigation to help understand the underlying cellular signaling it modulates.

Defined and temporal control of gene expression is very useful for understanding basic biological mechanism and medical research applications. The tetracycline (Tet)-regulated gene expression system is based on the regulatory elements of tetracycline – resistance operon of *E.coli.* The antibiotic tetracycline mediates “on/off” situation of gene activity by either promoting or preventing the binding of tet repressor (TetR) to the operator located in the promoter region. The Tet system reduces many of the pleiotropic effects of other inducible systems responsive to heavy metal ions, heat shock or hormones, which include leakiness, toxicity in mammalian cell culture, transgenic mice and other species [8], [9], [10], [11], [12], [13], [14]. To gain detailed insight into *E4orf1*-induced glucose disposal function and its therapeutic potential, here we describe the development of doxycycline-regulated 3T3-L1 cell line with inducible, stable expression of adenoviral *E4orf1* gene. We have also studied the kinetics of E4orf1 mediated glucose disposal in conjunction with its expression. Such an inducible system is highly suitable for investing how proximal insulin signaling may be bypassed to enhance cellular glucose uptake.

## Materials and Methods

### Cell culture and cell lines: Cell Culture

3T3-L1 cells were obtained from American Type Culture Collection (ATCC #CCL-92-1, Mannassas, VA) and maintained in high glucose Dulbecco's Modified Eagle Medium (DMEM) (Invitrogen, #11995), 10% normal calf serum (#SH30072.03, Hyclone) and an antibiotic-antimycotic agent (1%) (Sigma Aldrich #A5955).

3T3-L1 E4orf1 clones, when ready, were maintained in Tet-free fetal bovine serum (Clonetech, #631101) with 0.25 µg/mL puromycin and 0.05 µg/mL hygromycin (Invitrogen, #10687-010).

### Tet-On system

The Tet-On Advanced system was purchased from Clontech laboratories (Cat# 631059). This system is a complete inducible gene expression system including regulatory tTA vector (pTet-On), a response vector (pTRE-Tight) and a control vector (pTRE-Tight-Luc).

### Construction of the pTRE-Tight-*E4orf1* plasmid

Ad36 *E4orf1* DNA and amino acid sequence are previously reported [15], [16]. The full length 378bp cDNA of *E4orf1* was obtained by RT-PCR. The PCR primers were as follows: 5′-CCG AGA TCT ATG GCT GAA TCT CTG TAT GCT TTC-3′ and 5′-CGC GTC GAC CTA AAC CAG GGT GGC TAT TCT-3′, which contained restriction sites for BglII and SalI respectively. The resulting PCR products were further purified using QIAquick gel extraction kit (Qiagen, Cat # 28704) according to manufacturer's instructions. The full length *E4orf1* cDNA was cloned into pTRE-Tight vector using restriction enzymes BglII and SalI (New England Bio Labs).

### Transformation, purification, identification of plasmids and sequence analysis

The vectors provided withthe pTet-On system and the constructed recombinant pTRE-Tight-*E4orf1* were transformed into *E.coli* (DH5α). Plasmid DNA was purified using QIAprep maxi kit (Qiagen, Cat # 12362) according to the manufacturer's instructions. The purified plasmids and *E4orf1* cDNA were digested with the restriction enzymes BglII and SalI for sub-cloning and also to confirm the isolated clones for cDNA insert and the plasmid vector. The purified plasmid pTRE-Tight-*E4orf1* was sequenced with an ABI PRISM Big Dye Terminator cycle sequencing kit.

### Transfection of pTet-On plasmid

3T3-L1 cells were transfected with the pTet-On, pTRE-Tight-E4orf1 or pTRE-Tight-Luc plasmids using Lipofectamine reagent (Life Technologies, Cat #18324-012) according to the manufacturer's instructions. The transfected cells were selected in 1 µg/mL hygromycin for 2 weeks and the selection media was replaced every 4 days. Well-separated antibiotic resistant clones were individually picked with cloning discs and transferred to 24-well plates in selection medium. The cells were then transferred to larger culture vessels before confluence, and aliquots of early passages of cells were frozen in liquid nitrogen. A total of 22 clones were selected and expanded for screening.

### Screening for inducible puromycin-resistant clones by pTRE-Luc

All clones were plated in 12-well plates. The next day, at 50% confluence, clones were transfected with pTRE- Tight-Luc plasmid DNA by using Lipofectamine. Forty-eight hours post transfection; cells were exposed to 0, 0.1, 1, 10, 100, or 1,000 ng/mL of Doxycycline (Dox). Twenty-four hours post Dox-treatment, the luciferase activity was analyzed using luciferase assay system kit (Promega #1531). To further determine the optimal time point post Dox treatment and the effect of a range of Dox dose, the clone exhibiting maximum luciferase activity was then plated and again transfected with pTRE- Tight-Luc, and treated with 0, 100, 1,000, or 10,000 ng/mL Dox, two days post transfection. The luciferase activity was then determined at 6, 12, and 48 h. An un-transfected group was used to subtract background illumination.

### Transfection of pTet-On clone by pTRE-Tight-*E4orf1* plasmid

The pTet-On Advanced clone exhibiting maximum luciferase activity was split into 12-well plates for 50% confluency and transfected the next day with lipofectamine pTRE-Tight or pTRE Tight-*E4orf1* plasmid DNA. The clones were selected using 0.15 µg/mL of hygromycin for two weeks. Healthy hygromycin-resistant clones were individually picked with cloning discs and transferred to 24-well plates in selective medium. A total of 5 clones were selected and expanded for screening.

The cells were then transferred to larger culture vessels before confluence, and aliquots of early passages of cells were frozen in liquid nitrogen.

### E4orf1 clone characterization

The effect of Dox dose and treatment duration was determined by treating the clone exhibiting maximum *E4orf1* expression with1,000 ng/mL of Dox and RNA harvested 16 h, 24 h, or 48 h post treatment to determine *E4orf1* expression by RT-PCR.

#### Long term effect of inducible E4orf1 expression on glucose disposal

We determined the effect of *E4orf1* induction or withdrawal on glucose uptake. The 3T3-E4 inducible clone #1 and pTRE empty vector clones were induced with 1,000 ng/mL Dox for 24, 48, 72 or 96 h. In addition, we also determined the effect of withdrawal of Dox for 24 h after 96 h of induction, and reintroduction of Dox for 24 or 48 h. Cellular glucose uptake and *E4orf1* expression were determined.

### Techniques and assays

#### Quantitative real-time PCR

E4orf1 was detected using quantitative real time PCR (qRT-PCR). RNA was harvested and isolated using a RNA Mini Easy Kit (Qiagen, #74104), and cDNA was synthesized via RT PCR (Applied Biosystems # 4368814) according to manufacturer's instructions. An Ad36 *E4orf1* FAM nonflourescent primer-probe was custom designed and synthesized (Integrated DNA Technologies). The *E4orf1* primer probe combination is as follows: Probe- 5′-/56-FAM/TGC TGC TCT/ZEN/TTA ACC ACA CGG ACC G/3IABkFQ/-3′ Primer 1-5′-CCC TCG CGG ACATAC AAA A-3′ Primer 2-5′-GCC GGG AGA AGA CAT GAT CTC-3′ Fifty ng of cDNA was loaded per well in duplicate for detection of *E4orf1*. GAPDH (glyceraldehyde phosphate dehydrogenase) primer probe was used as a housekeeping gene (Applied Biosystems, assay ID# Mm99999915_g1), and 2 ng per well was loaded in duplicate for each sample. Taqman Universal PCR Mix was used for both genes according to manufacturer's instructions (Applied Biosystems #4304437). Expression was detected with the Applied Biosystems 7900 Sequence Detection System and the ΔΔCt method.

#### Fluorescence Microscopy

3T3-L1 *E4orf1* double stable inducible clone#1 and pTRE empty vector clone were plated in 35 mm glass bottom coverslip containing petri dishes. After 24 h of incubation at 37°C, both *E4orf1* and pTRE clones were induced with 1,000 ng/mL Dox for 24 h. Using 40× images were captured with Zeiss Axioskop fluorescence microscope.

#### Western Blotting

Cells were harvested in RIPA buffer supplemented with anti-protease (Sigma Aldrich, #P8340) and anti-phosphatase inhibitor cocktail (Thermo Scientific #78420). Protein concentration was determined by BCA assay. SDS-PAGE was performed with 30 µg protein loaded on a 15% gel and proteins were transferred to PVDF membrane. E4orf1 was detected with 1∶1000 dilution of custom made anti-rabbit polyclonal antibody (Proteintech Inc, IL).

#### Glucose Uptake

Cells in 12 well plates were serum starved for 2 h, and then washed twice with PBS before adding 450 µL KRP (136 mM NaCl, 4.7 mM KCl, 10 mM NaPO4, 0.9 mM CaCl2, 0.9 mM MgSO4). Two to three wells were treated with KRP plus 100 nM cytochalasin (Sigma Aldrich, #C6762) for subtraction of nonspecific glucose uptake. Fifty µL of 10× isotope solution was then added to each well for a final concentration of 100 nM cold 2-deoxy glucose and 0.5 µCi/mL [3H]-2-Deoxyglucose (PerkinElmer #NEC720A250UC) for 5 minutes. Immediately after the 5 minute incubation, cells were washed in ice-cold PBS. Next, 500 µL of 0.05% SDS was added to each well. After incubating cells for 30 min at 37°C, 450 µL of cell lysate was added to a scintillation vial and the remaining 50 µL was used for protein determination via the Bicinchoninic acid (BCA) assay. Samples were read on a Beckman scintillation counter the following day and readings were normalized to protein content of each well.

#### Statistical Analyses

All assays were performed with a minimum of three biological replicates. For glucose uptake assays, 8–12 biological replicates were used, and normalized to protein content. Group means were compared to test the hypotheses, by using a two-way Student's t-test. Significance was considered at p<0.05.

## Results

### Identification of recombinant pTRE-*E4orf1* plasmids by restriction enzymes

The sizes of pTet-on, pTRE-Tight, the recombinant pTRE-Tight-*E4orf1* plasmid and the cDNA fragment of *E4orf1* were 4.9 Kb, 4.1 Kb, 4.47 Kb and 0.37 Kb respectively. To confirm the correct insertion of the *E4orf1* cDNA fragment in the pTRE-Tight vector, the recombinant pTRE-Tight-*E4orf1* plasmid was cleaved with BglII and SalI generating fragments of 0.37 kb (*E4orf1* cDNA fragment) and 4.1 Kb (pTRE-Tight plasmid)(data not shown). The sequence and orientation of the inserted *E4orf1* cDNA in the pTRE-Tight plasmid was verified by sequencing.

### Selection-stable expression of 3T3-L1/Tet-On clones through transient transfection by pTRE-Tight-Luc

**Figure 1:**
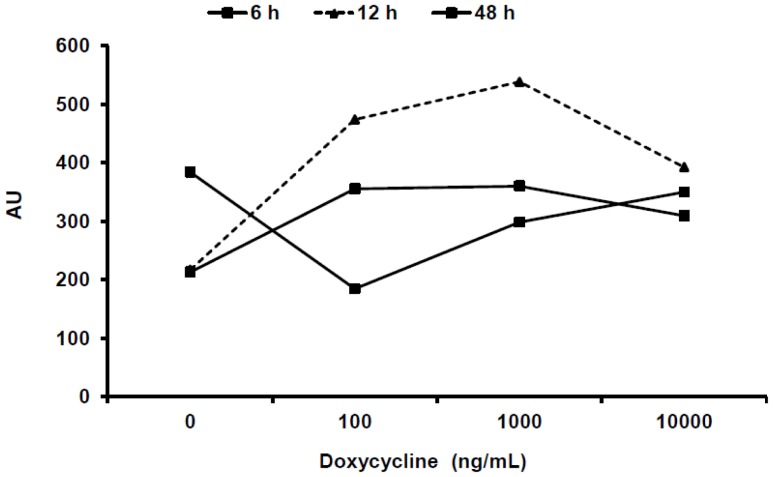
Transient transfection of 3T3-L1/Tet-On clone by pTRE-Tight-Luc and luciferase assay. 3T3-L1-pTet-On clone was transfected with pTRE-Tight-Luc plasmid. After 48 h post transfection, cells were treated with 0, 100, 1,000 ng/mL or 10,000 ng/mL of Dox and 6 h, 12 h and 48 h post treatment, the luciferase activity was analyzed using the luciferase assay system kit.

To establish a Tet-regulated inducible cell line, the pTet-On vector was first transfected into 3T3-L1 cells. Puromycin-resistant clones were selected and then transiently transfected with the pTRE-Luc plasmid. A total of 22 clones were selected and expanded for screening. Of the 3T3-L1-pTet-On clones selected and tested the inductive efficacy of doxycycline-regulated luciferase expression varied from 0 to 3 fold (data not shown). Out of all the clones, the clone which showed the highest level of luciferase expression was selected for further screening. To determine the best time point post Dox treatment and to determine the effect of a range of Dox treatments the selected clone was treated with 0, 100, 1,000, or 10,000 ng/mL Dox. The luciferase assay was then conducted at 6 h, 12 h, and 48 h post Dox treatment (Figure 1). At 12 h with 1,000 ng/mL Dox appears as the optimal dose with the highest luciferase expression (Figure 1).

### Identification of doubly-stable expression in 3T3-L1/pTet-On/pTRE-Tight-*E4orf1*

**Figure 2:**
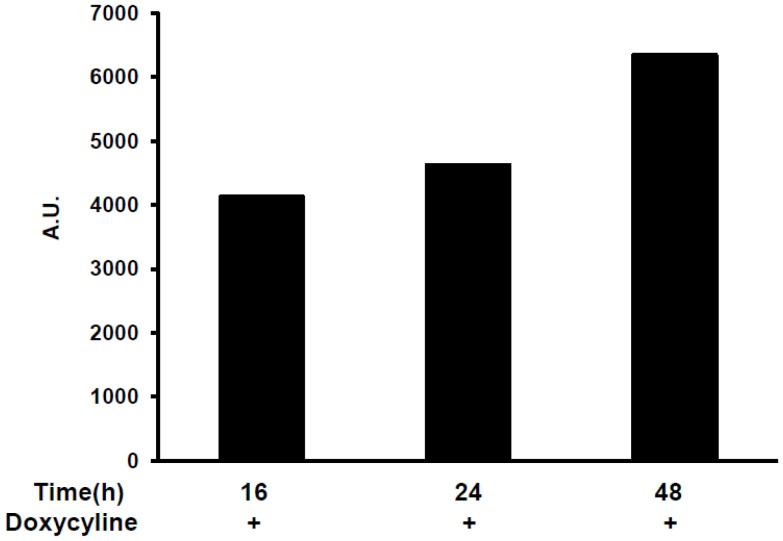
E4orf1 fold expression relative to pTRE. The pTRE TIGHT-E4orf1 clone was treated with 0 and 1,000 ng/mL Dox and RNA was harvested at either 16, 24 or 48 h post treatment. E4orf1 fold expression was determined relative to un-induced pTRE TIGHT-E4orf1 at 24 h using real time PCR assay.

The above described 3T3-L1-pTet-On clone exhibiting maximum luciferase activity was transfected with the recombinant pTRE-Tight-*E4orf1* plasmid. Hygromycin resistant 3T3-L1/pTet-On/pTRE-Tight-*E4orf1*clones were isolated and propagated. During screening the clone that showed the highest increase in *E4orf1* expression (6.5 fold) (data not shown), was selected for expansion and further characterization. The selected pTRE TIGHT-*E4orf1* was characterized for *E4orf1* expression by treating cells with 1000 ng/mL Dox, harvesting RNA after 16 h, 24 h and 48 h post Dox treament and determining *E4orf1* expression by RT-PCR. The *E4orf1* expression was determined relative to control 3T3-L1-pTet-On clone transfected with pTRE TIGHT-*E4orf1* vector and not induced with Dox (Figure 2). The selected *E4orf1* clone exhibited maximum *E4orf1* expression with 1,000 ng/mL Dox after 48 h induction (Figure 2).

**Figure 3:**
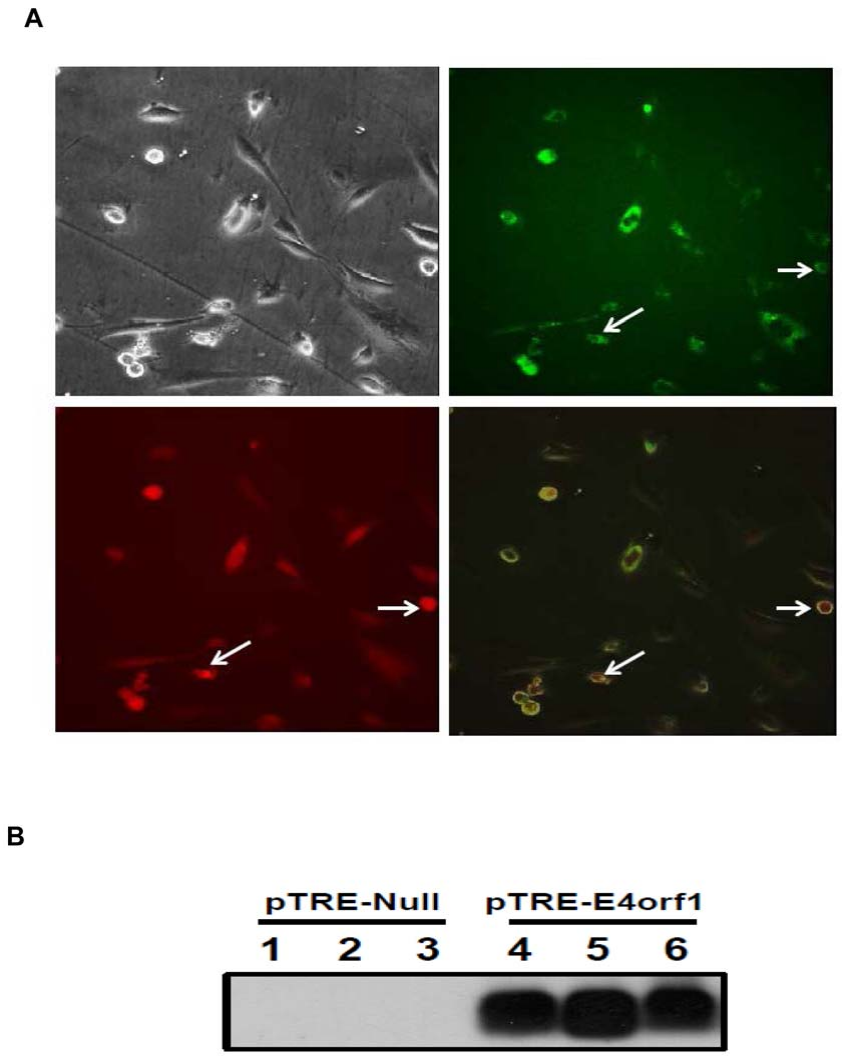
Stable expression of 3T3-L1/pTet-On and pTRE-Tight-E4orf1 plasmids and E4orf protein expression. **A**) Flourescence micrographs (40×) of live *E4orf1* double stable inducible clone induced with 1,000 ng/mL doxycycline for 24 h. Phase, p-Tet-On showing green fluorescence, p-TRE-Tight-E4orf1-Red fluorescence and Overlay. Arrows indicate co-localization. B) Western blot showing E4orf1 protein expression in pTRE and 3T3-E4 clone.

Additionally, fluorescence microscopy confirmed the dual stable transfection. The green fluorescence observed is exhibited by the p-Tet-On plasmid and red fluorescence is emitted by the recombinant pTRE-Tight-*E4orf1* plasmid (Figure 3A). There was no detectable *E4orf1* protein expression in pTRE null cells whereas very high protein expression was observed in the pTRE-Tight-*E4orf1* 3T3-E4 clone cells (Figure 3B). Stable *E4orf1* mRNA expression was determined up to 13 passages for the clone (data not shown).

#### Effect of regulated E4orf1 expression on glucose disposal

**Figure 4:**
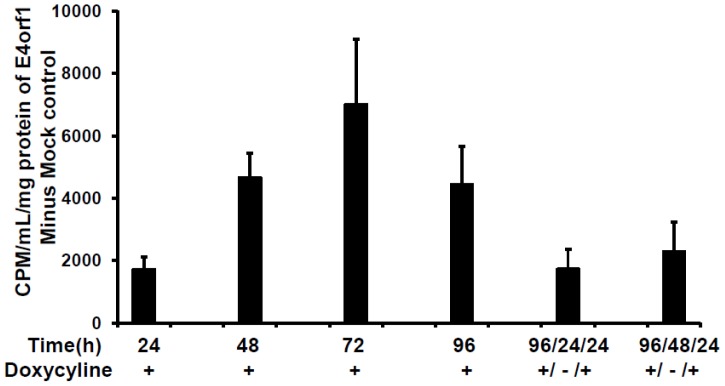
Effect of regulated *E4orf1* expression on glucose disposal. 3T3-E4 inducible clone and pTRE empty vector clones were induced with 1,000 ng/mL Dox for 24, 48, 72 and 96 h. After 96 h of induction, Dox was removed for 24 h or 48 h and reintroduced for 24 h or 48 h. At all-time points the glucose uptake in E4orf1 groups was significantly greater compared to the respective pTRE groups as determined by student T-Test (p<0.00001). Mean + SD. The difference of glucose uptake between pTRE and E4orf1 groups is presented, which was calculated by subtracting the average of the pTRE group values from the individual biological replicate value for the respective E4orf1 group.

To further elucidate the quantitative and temporal control of *E4orf1* gene expression on therapeutic potential to improve glucose disposal, 3T3-E4 inducible clone and pTRE empty vector clones were induced for variable time period. The glucose uptake at these time points is represented as the fold difference between pTRE empty vector and *E4orf1* groups (Figure 4).

**Figure 5:**
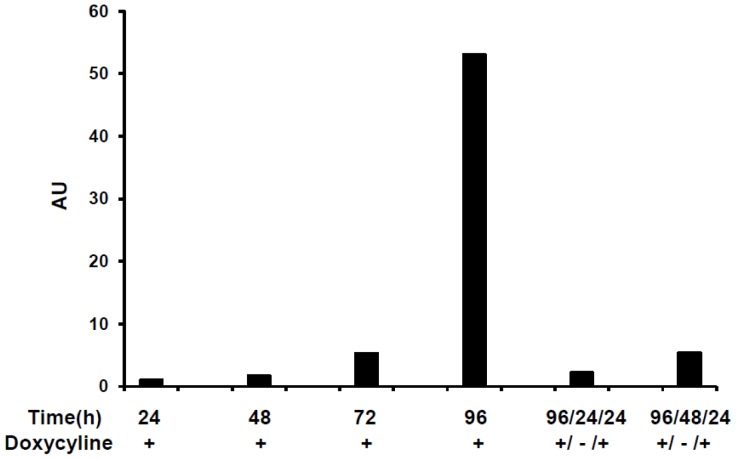
Regulated *E4orf1* expression at different time points of glucose uptake. 3T3-E4 inducible clone and pTRE empty vector clones were induced with 1,000 ng/mL Dox for 24, 48, 72 and 96 h. After 96 h of induction, Dox was removed for 24 h or 48 h and reintroduced for 24 h or 48 h. RNA was harvested and E4orf1 mRNA expression was determined using real time PCR. The E4orf1 mRNA is presented as fold difference compared with un-induced E4orf1 cells at 24 h.

This experiment had 6 conditions and 2 groups per condition (pTRE-null group and *E4orf1* expressing group). Under all 6 conditions, the 3T3-E4 groups had significantly greater glucose uptake vs their respective counterparts (p<0.00001 or better). The *E4orf1* induced glucose uptake appears to peak upon 72 h induction with Dox (Figure 4). To test the ability of the clones to respond to repeated induction, Dox was removed for 24 h or 48 h and later reintroduced for 24 h or 48 h. Reintroducing Dox did not improve *E4orf1* mediated glucose uptake (Figure 4), and glucose uptake levels were similar to the time period of the same duration as the reintroduction period. The *E4orf1* expression was confirmed in all groups using qRT-PCR (Figure 5), which peaked in response to 96 h induction with Dox.

## Discussion

Previous studies have identified the significance of Ad36 and its *E4orf1* protein in enhancing glucose disposal [17], [18], [19]. In addition, *E4orf1* of other adenoviruses is being extensively studied for its effect on cell polarity, tight junction and migration [20], [21]. Recently, a desirable role of exogenous *E4orf1* in primary endothelial cell survival was described, which allows in depth study of these cells, while preserving their vascular repertoire [22]. Considering the potential application of *E4orf1* in developing an anti-diabetes drug, or other important applications in understanding cell functions, a cell model is required to carefully study the interaction of E4orf1 with host signaling.

Until now, such a model was not available. Adipose tissue, adipocytes and their progenitors have been the main target of investigation for effects of *E4orf1* [7], [16], [18]. Therefore, considering the significance of adipocytes, here we developed a stable cell line using 3T3-L1 cells. This template could also be followed to develop other cell types that inducibly and stably express *E4orf1* of an adenovirus of interest. Importantly, several other adipogenic adenoviruses have been reported. The system we used may be employed to develop stable clones that would express *E4orf1* protein of other adenoviruses.

Ad36 infection of 3T3-L1 cells is abortive and the expression of *E4orf1* in Ad36 infected cells lasts for a short time, and is accompanied with the expression of other viral genes [23], which prevents conclusions about the exclusive *E4orf1* interactions with host cell signaling. *E4orf1* protein is a secreted protein and does not have a known receptor. Therefore, to study its action, it needs to be expressed intra-cellularly. While transient transfection of cells with *E4orf1* expressing plasmid is an option and exhibits a moderate degree of expression for 2 to 3 days. However, this method does not allow for careful dose-response analysis, and in addition, any effect of *E4orf1* is diluted since not all cells are successfully transfected. In addition, the transient transfection of cells is costly and time consuming. On the other hand, a cell line that stably expresses *E4orf1* can be used for many passages and allows easy derivation of activator cell line. Furthermore, an inducible system allows for accurate and quantitative analysis of the function of *E4orf1*. The limitations of this system include a low level of baseline *E4orf1* expression– a limitation common to many inducible cell systems. It should also be noted that these effects of E4orf1 are observed *in vitro*. *In vivo* effects and the significance of this protein needs to be determined.

The set of experiments presented here point to several guidelines for the use of this cell line. First, the highest *E4orf1* gene expression is obtained 96 h post dose of 1,000 ng/ml Dox. There appears to be a time x Dox-dose interaction (Figure 2). Thus, the Dox-dose for maximal expression may be considered, depending on the desired experimental design. Even though *E4orf1* gene expression levels are highest after 96 h of Dox treatment, glucose uptake was maximal after 72 h. This suggests a feedback mechanism may be induced after a certain threshold level of *E4orf1* protein has reached. At all time points studied, *E4orf1* significantly increased basal glucose uptake compared to empty vector when treated with 1,000 ng/ml of Dox, suggesting this is an optimal dose for studying the effect of *E4orf1* on glucose uptake.

To test the ability of the 3T3-E4 clone to respond to repeated induction, Dox was administered for 96 hours, then removed for either 24 or 48 h, and reintroduced for 24 h. Reintroducing Dox did not improve *E4orf1* mediated glucose uptake, or levels of gene expression. In fact, gene expression levels and glucose uptake were similar to that seen in the single 24-hour period. Thus, it appears that removing Dox for 24 hours or 48 hours may be sufficient to reduce *E4orf1* gene expression levels to baseline levels, and reintroduction of Dox for 24 hours produces a similar gene expression and glucose uptake effect as a novel exposure would. This indicates the cell line is flexible and consistent in its time course gene expression response to Dox.

This system offers considerable advantage, particularly to investigate cell signaling underlying *E4orf1*-induced glucose disposal. Based on the studies of Ad36 [24] and *E4orf1* of other adenoviruses [25], [26], the current working hypothesis is that *E4orf1* complexes with Dlg1 protein, which activates phosphatidyl inositol-3 kinase (PI3K) signaling via Ras activation, which leads to increased cellular glucose uptake [18]. This candidate pathway bypasses proximal insulin signaling, including insulin receptor and insulin receptor substrates (IRS), which is required to activate PI3K. *E4orf1* inducible cell line described here provides an opportunity to test an important template for proximal insulin signaling-independent glucose uptake, and should help in carefully elucidating signaling important in diabetes, and obesity.
